# Pre-Hospital Trauma Care in Road Traffic Accidents in Kashan, Iran

**DOI:** 10.5812/atr.8780

**Published:** 2013-02-01

**Authors:** Mohammad Paravar, Mehrdad Hosseinpour, Shayesteh Salehi, Mahdi Mohammadzadeh, Abolfazl Shojaee, Hossein Akbari, Azadeh Sadat Mirzadeh

**Affiliations:** 1 Faculty of Nursing and Midwifery, Khorasgan (Isfahan) Branch, Islamic Azad University, Isfahan, IR Iran; 2 Trauma Research Center, Kashan University of Medical Sciences, Kashan, IR Iran

**Keywords:** Accidents, Pre-hospital Care, Traffic, Wounds and Injuries

## Abstract

**Background:**

Iran has one of the highest rates of road traffic accidents (RTAs) worldwide. Pre-hospital trauma care can help minimize many instances of traffic-related mortality and morbidity.

**Objectives:**

The aim of this study was to assess the characteristics of pre-hospital care in patients who were injured in RTAs, admitted to hospital. The focus was mainly directed at evaluating pre-hospital trauma care provided in city streets and roads out of the city.

**Patients and Methods:**

This retrospective study was carried out on all trauma patients, transported by the emergency medical service (EMS) system, who were admitted to Kashan Shahid-Beheshti hospital during the period from March 2011 to March 2012. The patients’ demographic data, location of accident, damaged organs, mechanism of injury, injury severity, pre-hospital times (response, scene, transport), pre-hospital interventions and outcomes, were extracted from the data registry and analyzed through descriptive statistics using SPSS 18 software.

**Results:**

Findings of this study showed that, 75% of RTAs occurred on city streets (n = 1 251). Motor-car accidents were the most frequent mechanism of RTA on city streets (n = 525) (42%), while car rollover was the most frequent mechanism of RTA on roads out of the city (n = 155) (44.4%). The mean pre-hospital time intervals (min); response, scene, and transport for all patients were 6.6 ± 3.1, 10.7 ± 5 and 13 ± 9.8, respectively. The mean pre-hospital time intervals (response, scene, transport) in roads out of the city were higher than those in city streets. There was a significant difference (P = 0.04) in the mortality rates due to RTAs between city streets (n = 46) and roads out of the city (n = 32).

**Conclusions:**

In comparison with road traffic accidents on city streets, trauma patients in RTAs on roads out of the city have longer pre-hospital time intervals and more severe injuries; therefore, this group needs more pre-hospital resuscitation interventions.

## 1. Background 

The issue of road traffic accidents (RTAs), with 50 million injuries annually, is a major public health problem in developing countries (1). Iran has the highest rate of RTAs worldwide (2). After cardio- and cerebrovascular diseases, RTAs are considered to be the third leading cause of mortality in Iran. Each year approximately 28000 people are killed and 800000 (1.1% of the population) are injured on city streets and roads of Iran (2-4). Like many countries, the emergency medical service (EMS) is a system responsible for providing pre-hospital trauma care in Iran in 1977. In the EMS, emergency medical technicians (EMTs) are trained to provide basic life support (BLS) and advanced life support (ALS) for trauma patients. The US National Highway Traffic Safety Administration created the EMS-symbol or ‘Star of Life’ symbol which represents the six EMS functions for pre-hospital trauma care including; detection, reporting, response, on-scene care, care in transit and transfer to hospital (5, 6). The majority of RTA deaths in developing countries occur in the pre-hospital setting (7, 8). Studies in Iran have shown that about 60% of the deaths occurred at the crash scene or on the way to hospital (3). Pre-hospital care in RTAs increased dramatically form 7.5% to 60.4% during 2002-2011 in Iran (9, 10). To mitigate the consequences of RTAs, EMS capabilities in terms of human and physical resources have improved in recent years (11). In addition, improvements in pre-hospital care may decrease trauma mortality during the first few hours after injury, and it may also reduce the long-term mortality and morbidity rates due to RTAs (7, 12-14). Few studies have been done on pre-hospital trauma care for the injured people in Iran and those that have been conducted have mainly focused on evaluating pre-hospital trauma care provided in Tehran, which is a metropolitan city (11, 15, 16), while no studies have been carried out in other regions in Iran, especially cities located on main roads out of the city. We hope that this study results in improving the knowledge of pre-hospital trauma care, especially in RTAs in Iran.

## 2. Objectives

This study was designed to describe the characteristics of patients requiring pre-hospital care, which were injured in RTAs and admitted to hospital. The focus was mainly on evaluating pre-hospital trauma care provided on city streets and roads out of the city.

## 3. Patients and Methods

This retrospective study was performed on all trauma patients, conveyed by EMS, and admitted to hospital during March 2011 to March 2012. Data were extracted from a data bank of trauma research center and EMS registry data including; demographic data of patients, place of accident, damaged organs, mechanism, injury severity, pre-hospital times (response, scene, transport), pre-hospital interventions and outcomes. Patients who had injured resulted in immediate admission to hospital for two days or longer were included in the study and those who had died either at the scene or on route to the hospital excluded from the study. The pre-hospital time intervals were defined as the sum of the following time intervals including; response interval (time from alarm activation to arrival of the first responding vehicle on the scene) on-scene interval (time arrival of the first EMS response vehicle on the scene until leaving the scene) transport interval (time leaving the scene to the vehicle’s arrival at the receiving hospital).

Injury severity was defined using a revised trauma score (RTS). The RTS was calculated using the following formula; Glasgow Coma Score (GCS), systolic blood pressure (SBP) and respiratory rate (RR). RTS ranged from 0.00 to 7.84. Variables were described by the number of cases and percentages for the categorical data, and as means and standard deviations for the numerical data. The pre-hospital time intervals and interventions were set as independent variables. Dependent variables were classified into two groups, city streets vs. roads out of the city. Associations were analyzed using X^2^ tests. The statistical significance level was chosen as 5% (P = 0.05).

## 4. Results

The mean age of the patients was 34.4 ± 19.2 years (age range, 2-94 years) and the male: female ratio was 4.7:1. The mechanisms of injury were; motorcycle (59.2%), car (23.7%), pedestrian (15.8%) and bicycle (1.3%). In total, 25% of trauma patients (n = 400) had more than two injured organs. Head and neck injuries were the most common (52%) and injuries in the upper and lower extremities (49.7%) stood in the second place. Only 3.1% of motorcyclists had used a helmet, and 16.1% of car occupants had fastened their seat belt. The mean of the RTS in all patients was 7.46 ± 0.86. Table 1 shows the characteristics of trauma patients and pre-hospital care. The mean response time, at scene time, and transport time for all patients were 6.6 ± 3.1, 10.7 ± 5 and 13 ± 9.8, respectively. Intravenous (IV) access was established for all patients. Mean systolic blood pressure was 115.1 ± 17.7 mmHg. Four hundred and eleven (25.7%) patients were treated with IV fluids, and 344 patients (21.5%) had a Glasgow Coma Score (GCS) of less than 13. Spinal protective devices (neck collar and long backboard) were used for 39% of the cases. Thirty-one patients underwent endotracheal intubation (ETI) for airway protection at the crash scene or on the way to the hospital. Cardiopulmonary resuscitation (CPR) was performed on three injured patients (0.2%) at the accident scene. Mortality rate was 4.9% (n = 78).

**Table 1:** Characteristics of Trauma Patients and Pre-Hospital Care

Variable	Range	Mean ± S.D
**Age , y**	2 - 94	34.4 ± 19.2
**RTS a**	2.69 - 7.84	7.46 ± 0.86
**GCS a**	3 - 15	13.7 ± 2.5
**SBP, mmHg a**	60 - 200	115.1 ± 17.7
**DBP, mmHg a**	40 - 110	73.1 ± 9.8
**RR a**	5 - 30	16.2 ± 2.7
**PR a**	56 - 130	81.8 ± 9.9
**Response time, min**	1 - 36	6.6 ± 3.1
**On scene time, min**	3 - 40	10.7 ± 5
**Transport time, min**	2 - 120	13 ± 9.8
**Hospital stay**	1 - 50	5.5 ± 6.4

^a^Abbreviations: DBP, diastolic blood pressure; GCS, glasgow coma score; PR, pulse rate; RR, respiratory rate; RTS, revised trauma score; SBP, systolic blood pressure

Overall, 75.8% of RTAs occurred on city streets (n = 1251) and the remainder occurred on roads out of the city. Table 2 shows the characteristics of trauma patients on city streets and those on roads out of the city. According to the results in Table 2, there was no significant difference in the mean age of patients in city streets (34.1 ± 19.6) and roads (35.5 ± 17.8), while there was a significant difference in gender of the victims between the two groups of accidents (accidents on city streets and on roads out of the city) (P = 0.008). Motor-car accident was the most frequent mechanism of RTA on city streets (n = 525) (42%), while car rollover was the most frequent mechanism of RTA on roads out of the city (n = 155) (44.4%). Head and neck injuries (n = 209) (60%) mostly occurred on roads out of the city, while the majority of upper and lower extremity injuries (n = 705) (56.4%) occurred on city streets. There was a significant difference in the mortality rates between accidents on city streets (n = 46) and those on roads out of the city (n = 32) (P = 0.04).

**Table 2:** Characteristics of Trauma Patients in RTA

Demographics	Total	City Streets, No. (%)	Roads Out of The City, No. (%)	P Value
**Gender, No. (%)**				
Male	1323 (82.7)	1033 (85.2)	290 (75)	0.008
Female	277 (17.3)	180 (14.8)	97 (25)	
**Age, y, Mean ± SD**	34.4 ± 19.2	34.1 ± 19.6	35.5 ± 17.8	0.2
**Age range, y, No. (%)**				
Child (under 19)	348 (21.8)	292 (24.1)	56 (14.6)	0.01
Adult (20 – 59)	1013 (63.3)	732 (60.4)	281 (72.2)	0.01
Old (over 60)	239 (14.9)	188 (15.5)	51 (13.2)	0.59
**Mechanisms of injury, No. (%)**				
Car-car	187 (11.7)	115 (9.5)	72 (19)	0.004
Car rollover	191 (11.9)	18 (1.5)	173 (45)	< 0.001
Motorcycle-car	577 (36.1)	510 (42)	67 (17)	< 0.001
Motorcycle-motorcycle	221 (13.8)	178 (14.6)	43 (11)	0.33
Motorcycle- rollover	150 (9.4)	131 (11)	19 (5)	0.03
Pedestrian-car	118 (7.4)	110 (9)	8 (2)	0.003
Pedestrian- motorcycle	135 (8.4)	130 (10.7)	5 (1)	< 0.001
Bike	21 (1.3)	21 (1.7)	0 (0)	0.2
**Organ injuries, No. (%)**				
Multiple	408 (25.5)	300 (24.7)	108 (27.9)	0.51
Head and Neck	832 (52)	612 (50.4)	220 (56.8)	0.18
Chest	160 (10)	104 (8.5)	56 (14.4)	0.05
Abdomen	120 (7.5)	80 (6.5)	40 (10.3)	0.15
Limb	794 (49.6)	644 (53)	150 (38.7)	0.003
Pelvic	104 (6.5)	77 (6.3)	27 (6.9)	0.85
**Hospital stay, Mean ± SD**	5.5 ± 6.4	5.2 ± 5.6	6.5 ± 8.5	< 0.001
**Death, No. (%)**	78 (4.9)	46 (58.6)	32 (41.4)	0.04

The roads out of the city had significant RTS ≤ 4 (OR: 2.67, 95%, CI 0.8 - 8.89), ETI (OR: 2.07, 95%, CI 0.98 -4.37) and IV fluid administration (OR: 1.8, 95%, CI 1.2 - 2.8). Table 3 shows the pre-hospital time intervals and care administered on city streets and roads out of the city. Obviously, the mean pre-hospital time intervals (response, scene, transport) in roads out of the city were higher than those on city streets.

**Table 3:** Pre-Hospital Times and Care in RTA (City Streets vs. Roads Out of the City)

Variables	City Street	Roads Out of the City	P value
**Systolic BP, Mean ± SD a**	116.6 ± 16.9	111.9 ± 19.9	0.136
**Diastolic BP, Mean ± SD a**	73.5 ± 9.4	72.1 ± 11.3	0.04
**Heart rate, bpm, Mean ± SD**	81 ± 9.1	84.3 ± 11.8	< 0.001
**Respiratory rate, Mean ± SD**	16.3 ± 2.5	16.6 ± 3.3	< 0.001
**Glasgow Coma Score, Mean ± SD**	13.9 ± 2.3	13.1 ± 3.1	< 0.001
**Trauma Score, RTS, Mean ± SD**	7.53 ± 0.75	7.25 ± 1.1	< 0.001
**IV fluid, No. (%) a**	274 (22.6)	137 (35.4)	0.003
**Intubation, No. (%)**	51 (4.2)	32 (8.3)	0.08
**Response time, min, Mean ± SD**	5.8 ± 1.9	8.9 ± 4.5	< 0.001
**On scene time, min, Mean ± SD**	9.7 ± 3.5	13.8 ± 7.2	< 0.001
**Transport time, min, Mean ± SD**	9.6 ± 4.9	23.7 ± 23.8	< 0.001

^a^Abbreviations: BP, blood pressure; IV, intravenous; RTS, revised trauma score

## 5. Discussion

RTAs in Iran and other developing countries are the leading cause of morbidity and mortality in young men (under 40 years). In our study, the Mean ± SD age of patients was less than 40 years. This finding was also reported by Roudsari et al. (17, 18), Nazari et al. (19) in Iran and Durak et al. in Turkey (20). Motorcycles were responsible for the majority of road traffic crashes in our study accounting for 59.2% of cases. The findings of this study were in accordance with those reported by Markogiannakis et al. in Greece (21), Chalya et al. in Tanzania (22) and Fazel et al. in Iran (9). In addition, motorcycle events are the most common injury mechanism on city roads. Approximately one half of the motorcyclists had a traumatic brain injury (TBI). There was a significant relationship between head injury and death (P = 0.000). In our study, 90% of the motorcyclists who died had sustained a head trauma. Only 3.1% of motorcyclists had used a helmet for protection. On the other hand, car mishaps were the most common injury mechanism on roads out of the city; however, only 16.1% of car occupants had fastened their seat belt for injury protection. The helmet is a successful device for preventing injuries among motorcyclists and it reduces the fatal and serious head injuries between 20% and 45% (23). The Peden et al. study (1) showed that the use of a protective device (helmet, seat belt) resulted in a 40% - 50% total injury reduction. Unfortunately, despite law enforcement of using injury protective devices, seat belt and helmet use was rare (18), and needs mandatory legislation. The mean of RTS in all patients was (7.46 ± 0.86), and this was similar to the study of Markogiannakis et al. in Greece (21). The RTS was an important predictive scale for survival (20). Although 75.8% of all RTAs occurred on city streets, trauma severity and mortality rate in traffic accidents occurred on roads out of the city were higher than those on city street. Results of the study were in accordance with those of Nazar et al. study (19). Roads out of the city included; highways, freeways and roads with high speed vehicles that resulted in serious injuries and death. According to the comprehensive coverage guidelines of Iranian EMS, response time must be less than eight minutes in 80% of EMS operations on city streets and less than 15 minutes in 80% of EMS operations on roads out of the city (24). Response time in our study was 6.6 minutes which is satisfactory. The mean of the pre-hospital time intervals (response, at scene and transport) in accidents occurred on city street were significantly lower than those in accidents occurred on roads out of the city. The results of this study were in accordance with those of the Bigdeli et al. study (25). In the Gonzalez et al. study, they showed that a pre-hospital time increase resulted in higher mortality rates (26). Our findings were in accordance with those of the Gonzalez et al. study. Fifty eight patients (9.7%) had a SBP less than 90 mmHg. A significant relationship was seen between SBP ≤ 90 mmHg with death (P = 0.000) and RTAs on roads out the city (P = 0.02) in our study. The results of Edelman et al. study (27) showed that trauma patients with a SBP ≤ 109 mmHg were at increased risk for morbidity and mortality following trauma. According to the results of Hasler et al. study (28), mortality rate was doubled in patients with SBP 90 – 109 mmHg, four-fold higher at 70 – 89 mmHg and 10-fold higher at < 70 mmHg compared to patients with SBP 110 – 129 mmHg. We suggest that all RTA trauma patients with a SBP less than 90 mmHg should be considered as a special group requiring aggressive resuscitation with fluid administration. The mean GCS of the patients who underwent airway intubation was 5.8 ± 1.8. Although an intubation improves patient outcomes (29), they need more time to perform. Therefore, some studies have shown that patients intubated at the scene may have unrecognized episodes of hypoxia or hypo-tension, thus leading to poorer outcomes (30, 31). Based on NAEMT (32) in situations such as out of city roads, and suburban locations with longer transport times, intubation may be more beneficial than no intubation at all, even when done by a less experienced operator. Our study showed that there was a significant relationship between airway intubation and survival rate (P = 0.003) in RTAs (city streets and roads out of the city). Moreover, our study suggests that all patients with severe head trauma (GCS ≤ 8) should be intubated. In conclusion, trauma patients on roads out of the city have a longer pre-hospital care interval and they also have more severe injuries. Therefore, this group requires more pre-hospital resuscitation interventions (eg, IV fluids and ETI).
